# Is Propolis a Potential Anti-Obesogenic Agent for Obesity?

**DOI:** 10.1007/s13668-024-00524-0

**Published:** 2024-03-04

**Authors:** Nilüfer Acar Tek, Şerife Akpınar Şentüre, Nursena Ersoy

**Affiliations:** 1 Faculty of Health Science, Department of Nutrition and Dietetic, Gazi University, Emek, Bişkek Main St. 6. St No: 2, 06490 Çankaya Ankara, Turkey; 2 Faculty of Health Science, Department of Nutrition and Dietetic, Ankara University, Fatih Caddesi No:197/7 PK:06290, Keçiören Ankara, Turkey

**Keywords:** Propolis, UCP-1, Creatinine kinase, Thermogenesis

## Abstract

**Purpose of Review:**

Propolis is a bee product that has been used for thousands of years. The chemical composition and biological activity of propolis, which has been investigated in the twentieth century, may vary according to location. Propolis polyphenols can induce thermogenesis in brown and beige fat tissue via the uncoupled protein-1 and creatinine kinase metabolic pathways. This review provides a comprehensive investigation of the structural and biological properties of propolis and provides insights into their promising potential strategies in body weight management.

**Recent Findings:**

By raising overall energy expenditure, it might lead to body weight management. Furthermore, the phenolic components artepillin C, quercetin, catechin, and chlorogenic acid found in its composition may have anti-obesogenic effect by stimulating the sympathetic nervous system, enhancing browning in white adipose tissue, and triggering AMP-activated protein kinase activation and mitochondrial biogenesis.

**Summary:**

Propolis, a natural product, is effective in preventing obesity which is a contemporary pandemic.

## Introduction

Propolis, often known as “bee glue,” was known and used for millennia by the Ancient Greeks, Romans, and Egyptians. Propolis is made by honey bees from diverse portions of plants and is made up of roughly 50% resin, 30% beeswax, 10% essential/aromatic oils, 5% pollen, and 5% different components [1]. Propolis comes from various locations and is classified into three types based on its predominate color: green, red, or brown [2••]. Propolis’ chemical composition was first studied in the early twentieth century, and it turned out that it contains more than 300 bioactive elements, particularly phenolic compounds [3, 4•].

Propolis formulations’ chemical composition and biological capabilities may differ depending on geographical region, climate, and plant source [2••, 4•]. As a result, the makeup of various propolis species, such as Brazilian, Chinese, and Anatolian propolis, differs [5]. The total flavonoid content of Anatolian propolis from different locations was reported to be between 16.13 and 199.69 mg/g [6, 7]. According to research investigating the contents of propolis obtained from various locations of Turkey, the phenolic composition of propolis consists of catechins, caffeic acid, chlorogenic acid, myristine, apigenin, and galangin [8–14].

Although propolis has been highlighted for its ability to strengthen the immune system through the COVID-19 process, it is also responsible for a wide range of antiviral, anti-inflammatory, and antioxidant activities due to polyphenols in its composition such as catechins, caffeic acid, Artepillin C, quercetin, and chlorogenic acid [15–17]. Various research has demonstrated that phenolic components such as catechin, quercetin, and chlorogenic acid have been associated to energy expenditure and lower body mass index (BMI) [18–22]. Polyphenols may have a role in body weight management by activating-oxidation, inducing satiety, increasing energy expenditure, promoting adipocyte death, and stimulating lipolysis [23, 24].

Obesity, which comes as a result of a malfunction in the management of body weight, is an important public health problem that is becoming more prevalent by the day. Obesity, which can evolve due to a variety of etiologies, is significant because of the comorbidities it causes and its potential to raise the risk of death rather than esthetic problems [25, 26]. Today, a rise in high-energy-dense food consumption and sedentary lifestyle causes energy intake and expenditure to shift towards energy intake, which points to the basic principle of positive energy balance in the formation of obesity [27].

While the oxidation of macronutrients in consumed foods provides energy intake, the energy consumed in this energy production process is referred to as the thermic effect. The components of energy expenditure include basal metabolic rate, thermic effect, and physical activity [28]. In the prevention and treatment of obesity, the balance of energy intake and expenditure is critical. Adipose tissue is a vital organ that actively contributes to the maintenance of this balance. While white adipose tissue stores energy in positive energy balance, brown adipose tissue transfers this energy via uncoupled respiration, which is mediated by uncoupled protein-1 (UCP1). Browning occurs when white adipose tissue acquires brown adipose tissue features through food consumption, as well as different environmental influences such as temperature change, physical exercise, and peroxisome proliferator-activated receptor gamma (PPAR-). Because of the increase in UCP-1 mRNA concentrations during the browning process of white adipose tissue, thermogenesis is triggered in the development of beige adipose tissue, and thus, energy expenditure increases [29]. In addition to UCP-1, the creatinine kinase manner has been identified as an additional pathway in the stimulation of thermogenesis in brown and beige adipose tissue. As with the UCP-1 pathway, increased creatine kinase enzyme expression in response to diverse environmental conditions catalyzes the conversion of ATP and creatine to phosphocreatine and allows energy to be dissipated as heat [30, 31]. Increased concentrations of brown and beige adipose tissue may enhance energy expenditure and so counteract obesity and its metabolic consequences [32].

Given these findings, particular nutrients that promote browning of white adipose tissue may hold promise for the prevention and treatment of obesity by increasing energy expenditure. Furthermore, certain nutrients, lipid metabolism, and interaction with adipose tissue can all have anti-obesogenic effects by influencing various enzyme expressions. Propolis is a natural apitherapy product that contains these chemicals and is a potential agent. According to reports, the population in ever-developing countries is increasingly turning to natural goods as a therapeutic option [33]. The purpose of this review is to discuss the effectiveness of propolis, a widely used and natural bee product, on energy expenditure.

## Metabolic Pathways of Weight Management

**Fig. 1:**
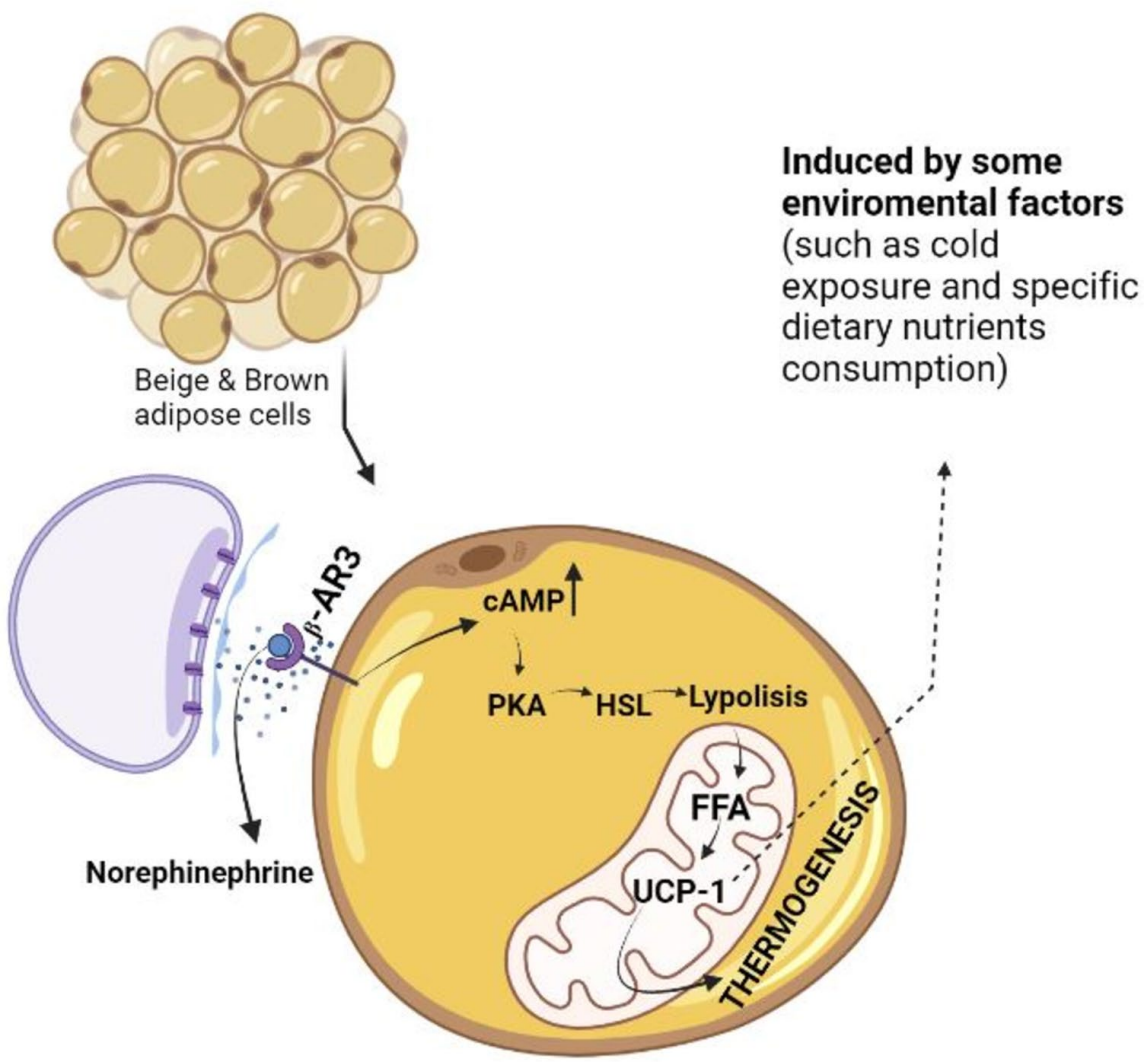
UCP-1 mechanism in energy expenditure. cAMP, cyclic adenine mono phosphate; β-AR3, β-Adrenergic receptor 3; PKA, Protein kinase; HSL, Hormone-sensitive lipase; UCP-1, uncoupled protein 1

Body weight management, or the achievement and/or maintenance of a healthy body weight, is critical in the prevention and treatment of obesity [34]. This process involves several metabolic pathways. UCP-1 is an essential mitochondrial membranous protein involved in the thermogenic process, particularly in brown adipose tissue (BAT). The sympathetic nervous system regulates UCP-1 expression via multiple methods that induce noradrenaline [35]. Binding of noradrenaline to the adipocyte plasma membrane’s 3-adrenergic receptor (3AR) is a result in increased intracellular cyclic adenosine monophosphate (cAMP) levels, cAMP-dependent protein kinase (PKA), and cAMP-sensitive element-binding protein (CREB) activation. P PKA activation increases the production of hormone-sensitive lipase (HSL), which promotes lipolysis, and the free fatty acids produced serve as substrates for BAT thermogenesis. UCP-1 is primarily generated in BAT via the activation or overexpression of many important biomolecules, including 3AR, peroxisome proliferator-activated receptor gamma coactivator 1-alpha (PGC-1), and PPAR-γ [36]. However, UCP-1 expression has been observed in white adipose tissue of rats in response to diverse stimuli [37]. UCP-1 is also detected in beige adipocytes produced in white adipose tissue (WAT) [29]. When environmental variables such as cold or food components activate UCP1, ATP phosphorylation is halted due to a break in the electrochemical gradient that stimulates ATP production, and hence respiratory chain activity is enhanced. Heat is produced by the burning of existing substrates and dispersed throughout the body via circulation [38]. As a result, UCP-1 expression helps to limit body fat storage by increasing energy expenditure [39•]. Figure 1 illustrates details on the mechanism of UCP-1. UCP-1’s effect on thermogenesis is critical for body weight regulation. Nutritional components help to manage body weight by increasing thermogenesis and total energy expenditure by activating UCP-1 and/or contributing to browning in adipocytes. Essentially, the amount of brown adipose tissue in which UCP-1 is expressed diminishes with age. As a result, emerging research suggests that specific dietary interventions may play a crucial role in promoting the development of beige adipocytes within white adipose tissue, offering potential avenues for addressing obesity [40]. Recent studies, such as Wang et al., have indicated a correlation between certain dietary components and the increased formation of beige adipocytes, presenting promising prospects for obesity management [41].

**Fig. 2:**
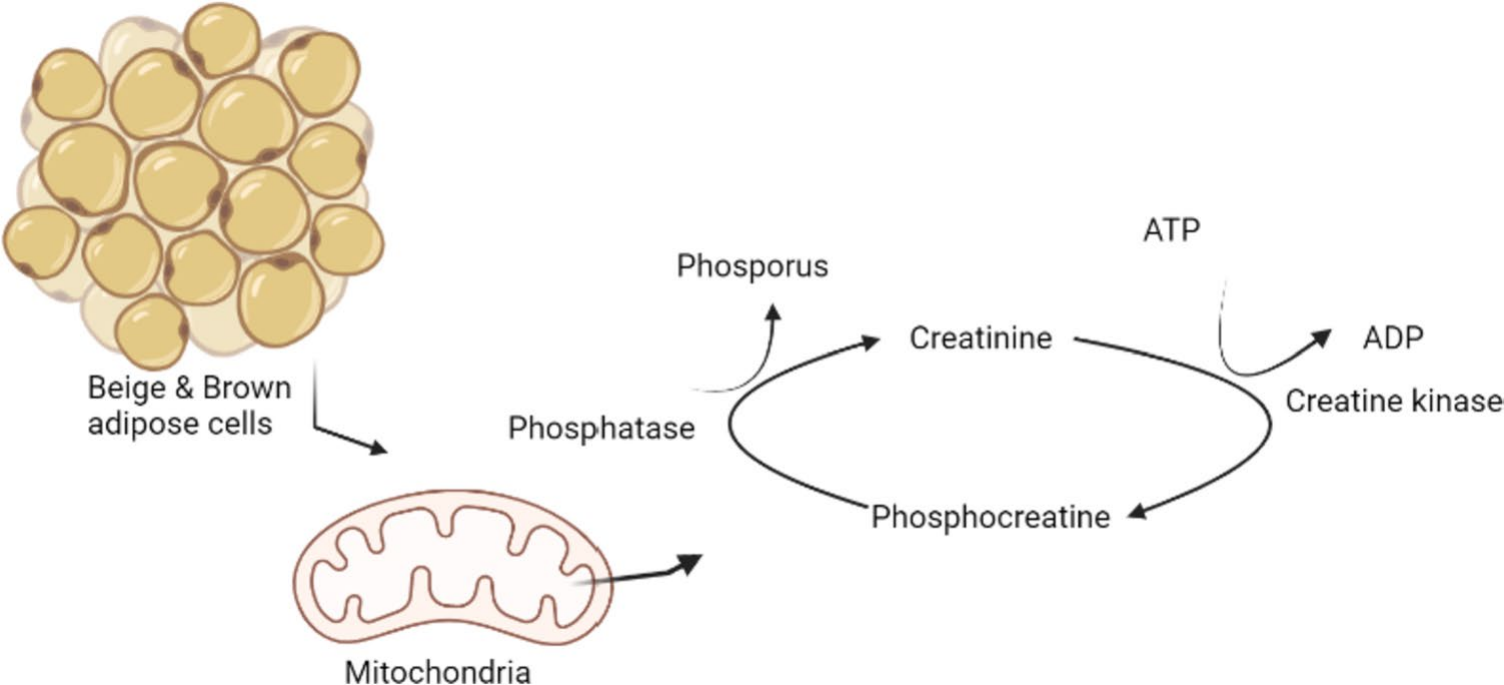
Creatinine cycle for energy expenditure

In addition to the UCP-1 pathway, the creatinine cycle is present in brown and beige adipocytes in adipose tissue to release chemical energy in the form of heat without mechanical or chemical activity. These cycles increase energy expenditure through thermogenesis [42]. The reversible transfer of a phosphoryl group from ATP to creatine is catalyzed by creatine kinases. In mammals, it is encoded by four different genes that demonstrate tissue-specific expression and variable subcellular localization. Creatine kinases located near ATPs created by selective oxidative phosphorylation release energy through thermogenesis by forming phosphocreatine with phosphorus acquired from ATP, particularly in brown and beige adipose tissue mitochondria. The phosphatase enzyme also degrades phosphocreatine and transforms it into creatinine to produce new ADPs. In fact, this transformation occurs in many tissues, but the ratio of mitochondrial creatinine kinase to total cellular creatinine kinase pool is higher in brown adipocytes than in other tissues, and the regulation of intracellular activation of the creatine kinase enzyme in these tissues can be done by a single isoenzyme [42–44]. As a result, the creatinine cycle in brown and beige tissue is critical for thermogenesis and energy expenditure. Details of this mechanism are presented in Fig. 2.

In addition to UCP-1 and cretin kinase pathways, there are additional lipolysis induction, lipogenesis, and adipogenesis inhibition processes that contribute to weight management. Lipolysis is induced by an increase in cAMP levels and HSL activity. Reduced fatty acid synthetase and acetyl-CoA carboxylase gene expression levels inhibit lipogenesis, while decreasing PPAR-γ and CCAAT/enhancing binding protein (C/EBP) gene expression levels inhibit adipogenesis [45].

## The Relationship of Polyphenols in the Composition of Propolis and Anti-Obesogenic Pathways

### Artepillin C

Artepillin C (ArtC), a cinnamic acid derivative, is a key component of propolis due to its high phenolic content. Several investigations have demonstrated that ArtC, particularly in Brazilian propolis, has antioxidant, anti-inflammatory, anti-diabetic, neuroprotective, gastroprotective, anticancer, and immunomodulatory properties [46]. ArtC has also been demonstrated to enhance beige adipose tissue and thermogenesis. ArtC induces beige adipocytes in murine C3H10T1/2 cells and primary inguinal WAT (iWAT) adipocytes; when orally administered at a dose of 10 mg/kg for 4 weeks, ArtC promotes the generation of beige adipocytes in mouse iWAT through increased UCP-1 expression [47•]. However, further studies are needed to elucidate its net effect on human metabolism. Artepillin C has been shown to stimulate thermogenesis by acting as a PPAR-agonist, as well as enhancing UCP-1 expression and creatinine metabolism [48]. Aside from these mechanisms, in vivo investigations have revealed that propolis possesses anti-obesogenic properties. Brazilian propolis consumption (100 mg/kg for 12 weeks) has been demonstrated to diminish visceral adipose tissue mass in obese mice [49]. Propolis interventions at various doses and times (50 mg/kg-10 days; 20 mg/kg-12 weeks) have been demonstrated to dramatically reduce body weight gain and accumulation of body fat in mice fed a high-fat diet [50, 51]. These findings indicate the anti-obesogenic activity of several phenols present in propolis by inducing BAT thermogenesis, sympathetic nervous system stimulation, browning in WAT, AMPK activation, and mitochondrial biogenesis [52].

### Quercetin

Quercetin is one of the most abundant polyphenols found in diet. Quercetin has been demonstrated to stimulate AMP-activated protein kinase (AMPK) and promote UCP-1 and enhance energy expenditure [53, 54]. Quercetin use (100 mg/day for 12 weeks) has been proven to reduce total body fat and BMI values in overweight/obese individuals [55]. Another study found that quercetin (150 mg/day) significantly reduced waist circumference in obese people [56]. Furthermore, it was underlined that the decrease in body weight and body fat percentages of obese patients following onion extract consumption was associated with quercetin [20]. It is emphasized that the anti-obesogenic activity of quercetin is related to BAT thermogenesis, browning in WAT, and mitochondrial biogenesis pathways, and the need for further research is underlined to clarify the mechanisms of its bioactive effects and bioavailability [54].

### Chlorogenic Acid

Chlorogenic acid is a polyphenol derived by the esterification of caffeic acid, a cinnamic acid derivative, with quinic acid. In an in vivo study examining chlorogenic acid, it emerged that obesity caused by high-fat feeding resulted in body weight loss with increased energy expenditure after 3 weeks of high fat and chlorogenic acid administration (0.21 mg/day decaffeinated phenol-chlorogenic acid, 2.3 g/day chromium dinicocysteinate, and 0.1 mg/day caffeine) [57]. In another in vivo investigation demonstrated that chlorogenic acid (100 mg/kg/day for 8 weeks) can reduce visceral fat accumulation, abdominal circumference, and body weight in rats fed a high-fat high-carbohydrate diet (16 weeks) [58]. A clinical investigation found that consuming beverages containing chlorogenic acid (600 mg/day chlorogenic acid-5 days) improved fatty acid oxidation in individuals with normal body weight [59]. In a different study, it was shown that oxygen consumption, fat use, and anaerobic threshold during exercise increased as a result of consumption of chlorogenic acid-containing beverages (359 mg/day chlorogenic acid-7 days) in individuals with normal body weight [60]. In a case (369 mg/day) control (35 mg/day) study conducted on overweight individuals and examining the effects of 12 weeks of chlorogenic acid given with coffee, it was found that body weight, abdominal fat area, visceral fat area, and waist circumference were significantly reduced in the intervention group compared to the control group [61]. Another study applying the same dose (369 mg/day for 7 days) found that it increased post-prandial fat utilization and energy expenditure [62]. It is suggested that these findings are related to a decrease in the expression of enzymes involved in fatty acid synthesis and the production of malonyl CoA and also the induction of energy expenditure by increasing fatty acid oxidation in mitochondria [63]. In addition to these mechanisms, in vivo and in vitro studies have shown that it is also effective on thermogenesis. In a study in mice, it was shown that chlorogenic acid (100 mg/kg-13 weeks) increased thermogenesis by increasing brown adipose tissue activity [64]. In addition, in an in vitro study, it was reported that both separately and in combination treatments of caffeic acid and chlorogenic acid dose-dependently induce browning in white adipose tissue, and they provide this function via AMPK activation and PPAR-γ modulation [65]. PPAR-γ expresses an important pathway in immune modulation, and it is reported that it can improve the functions of adipocytes and thus may be effective in obesity and metabolic syndrome [66].

### Catechins

Catechins are polyphenols reported to have anti-obesogenic properties with a pronounced thermogenic effect. Catechins are polyphenols that have been claimed to have strong thermogenic and anti-obesogenic effects. Activation of PGC-1-dependent pathways, norepinephrine/catechol-O-methyl-transferase (NE/COMT) inhibition, and mitochondrial biogenesis activation have all been linked to catechins’ ability to influence thermogenesis [67, 68]. By inhibiting COMT, a protein that is present in almost all tissues, catechins have been shown to stop the breakdown of catecholamines like norepinephrine. This allows the sympathetic nervous system to be continuously stimulated, increasing fat oxidation and energy expenditure through adeno-receptors [69]. However, there are inconsistent findings in the literature regarding this particular aspect in human studies [70].

The effectiveness of catechins on energy expenditure was assessed in the first study exploring the relationship between obesity and polyphenols, and it was discovered that consuming of green tea rich in catechins (50–90 mg) enhanced energy expenditure and fat oxidation [71]. Long-term catechin consumption, measured twice a day for 5 weeks, promoted thermogenesis activation with higher BAT activity [72]. In an in vivo investigation, tea catechin extract (100 mg/kg/day) was found to activate brown adipose tissue while also browning white adipose tissue [73]. Consumption of 1 bottle of oolong tea (690 mg catechin for 12 weeks) was found in a clinical research to reduce body weight, BMI, waist circumference, body fat mass, and subcutaneous fat ratio in healthy men [74]. Another study found that consuming catechin-containing beverages (609.3 mg catechin and 68.7 mg caffeine daily for 12 weeks) reduced body weight, visceral fat area, and body fat in individuals with abdominal obesity [75]. Although the results of these studies are thought to be due to caffeine’s positive effect, in vivo studies have shown that decaffeinated green tea catechins (7.7 g/kg/day) increase energy expenditure by stimulating thermogenesis and browning of white adipose tissue [76]. Enrichment of the diet with 1.0% of the catechin derivatives, epigallocatechin gallate (EGCG) for 4 weeks, enhances BAT thermogenesis and prevents obesity caused by a high-fat diet [68]. Consumption of EGCG (0.2% of the diet for 8 weeks) enhanced body temperature and gene expressions associated to thermogenesis in obese mice [77]. These effects have been linked to the activation of adenosine monophosphate-activated protein kinase (AMPK), which, by upregulating antiadipogenic genes including UCP-1, facilitates the browning of white adipocytes [78, 79].

## Conclusion

The prevalence of obesity is increasing day by day, and drugs and surgical methods developed for its treatment can lead to serious side effects. Therefore, alternative “natural” options, including propolis, which is on the rise. Various polyphenols have shown effects on thermogenesis, browning, and fat metabolism in body weight control. Similarly, propolis may be a natural anti-obesogenic agent in the prevention and treatment of obesity thanks to its high concentration of polyphenols.

Promising studies showing the effect of ArtC, quercetin, chlorogenic acid, and catechins in the composition of propolis on energy expenditure suggest that propolis may have beneficial effects for body weight control. Research on the bioactive effects and bioavailability of these components is essential. Since the composition of propolis varies according to its species, the effect of different species on energy metabolism will vary. Therefore, randomized controlled clinical trials examining the effect of different propolis species are needed. In light of current knowledge, according to EFSA recommendations, 750 mg/day propolis, which is a safe dose for adults, should be consumed [4•]. However, for the effect of propolis especially on energy metabolism, the recommended amount of propolis should be determined based on the effective phenolic component doses to be determined by clinical studies in the future.
